# Accounting for Viscoelasticity When Interpreting Nano-Composite High-Deflection Strain Gauges

**DOI:** 10.3390/s22145239

**Published:** 2022-07-13

**Authors:** Spencer A. Baker, McKay D. McFadden, Emma E. Bowden, Anton E. Bowden, Ulrike H. Mitchell, David T. Fullwood

**Affiliations:** 1 Department of Mechanical Engineering, Brigham Young University, Provo, UT 84602, USA; sbaker29@byu.edu (S.A.B.); mdm9333@byu.edu (M.D.M.); eebowden@byu.edu (E.E.B.); abowden@byu.edu (A.E.B.); 2 Department of Exercise Sciences, Brigham Young University, Provo, UT 84062, USA; rike_mitchell@byu.edu

**Keywords:** high-deflection strain gauges, nanocomposites, modeling, viscoelasticity

## Abstract

High-deflection strain gauges show potential as economical and user-friendly sensors for capturing large deformations. The interpretation of these sensors is much more complex than that of conventional strain gauges due to the viscoelastic nature of strain gauges. This research endeavor developed and tested a model for interpreting sensor outputs that includes the time-dependent nature of strain gauges. A model that captures the effect of quasi-static strains was determined by using a conventional approach of fitting an equation to observed data. The dynamic relationship between the strain and the resistance was incorporated by superimposing dynamic components onto the quasi-static model to account for spikes in resistances that accompany each change in sensor strain and subsequent exponential decays. It was shown that the model can be calibrated for a given sensor by taking two data points at known strains. The resulting sensor-specific model was able to interpret strain-gauge electrical signals during a cyclical load to predict strain with an average mean absolute error (MAE) of 1.4% strain, and to determine the strain rate with an average MAE of 0.036 mm/s. The resulting model and tuning procedure may be used in a wide range of applications, such as biomechanical monitoring and analysis.

## 1. Introduction

It has been postulated that surface-mountable high-deflection strain gauges can provide an economical and user-friendly solution in myriad motion-capture applications, such as human biomechanics and soft robotics. Conventional strain gauges can only endure approximately 5% strain, compared with potential skin strains of larger magnitudes; however, recently developed strain gauges, composed of conductive nanoparticles embedded in a silicone matrix, perform well at higher strains [1]. These sensors operate on the principles of quantum tunneling and percolation theory [2]. As the strain gauges are stretched, their electrical resistance decreases, causing the sensors to behave as inverse-piezoresistive sensors. These sensors can also display a high sensitivity factor in the desired range of deformation during a typical quasi-static response [3,4].

Interpreting the sensor output during multiple cyclic strains, however, is more complex than analyzing the output during quasi-static behavior. Due to the viscoelastic mechanical properties of strain gauges, the gauge factor is dependent not only on the instantaneous strain, but also on previously applied strains, as well as on the recovery time after those strains are applied [2]. The purpose of this research endeavor is to discover the time-dependent relationship between sensor strain and electrical response, accounting for the relevant viscoelastic mechanical properties of strain gauges. The uncovered relationships were captured in an appropriate model, and the model’s performance during cyclical stresses and relaxations was assessed.

Since deformation within the strain gauge largely occurs in the polymer matrix, it can be assumed that a standard rheological model captures their mechanical response and provides valuable insights regarding the electrical behavior. Several models have been postulated to capture viscoelastic mechanical behavior. The Burger’s model depicts the relationship between the strain, strain rate, and resulting stress of viscoelastic materials; and Weibull’s model predicts the strain relaxation of sensors after stresses are removed [5,6,7]. These two models provide valuable insight regarding the electrical behavior of sensors under dynamic loading and relaxation conditions.

One application that motivates the current study is to detect and quantify spinal biomechanics by adhering an array of such sensors to a subject’s lower back. The results of this study potentially offer valuable insights, suggesting that the method presented can be used to interpret sensor output when utilizing the strain gauges as a diagnostic tool. For example, the most common diagnostic methods for identifying the source of low-back pain employ static imaging (e.g., CT or MRI scans) to detect anatomical anomalies. It is difficult to pinpoint the etiology of chronic low back pain (cLBP) [8] using these methods because cLBP patients do not always exhibit clear anatomical anomalies in their spine [9]. Rather, their pain is the result of multiple psychological, social, and biological factors [10] that cannot be depicted on a scan. An alternative to using static diagnostic imaging is to analyze a patient’s spinal motions. To execute this alternate method, a valid and reliable quantitative assessment tool is necessary in order to objectively assess and track spinal health [11]. Such a tool could be used for the objective monitoring of patient progress, i.e., improved movement patterns. In addition, it could also lead to the discovery of more efficient treatment paradigms for the millions of chronic low-back pain patients worldwide. Furthermore, the information gleaned from the viscoelastic characterization of the sensors is relevant to the application of strain gauges in other fields, such as applications in soft robotics, human–machine interfaces, and other biomechanical analyses [1,12,13,14,15].

## 2. Materials and Methods

The nanocomposite sensors were composed of Ecoflex 00-30 silicone with nickel-coated carbon fibers (NCCFs) and nickel nanostrands (NINs) mixed in the matrix. Ecoflex 00-30 silicone was purchased from Smooth-On Inc., and the nanoparticles were purchased from Conductive Composites. The concentration of each material (by weight) for the sensor recipe was 64% silicone, 30% NCCFs, 5% NINs, and 1% surfactant. The nanoparticles and surfactants were mixed in the silicone matrix, then extruded into molds. The sensors were cured at 88 °C under a vacuum pressure of 650 mmHG for 135 min, and then at 192 °C for an additional 30 min.

**Figure 1:**
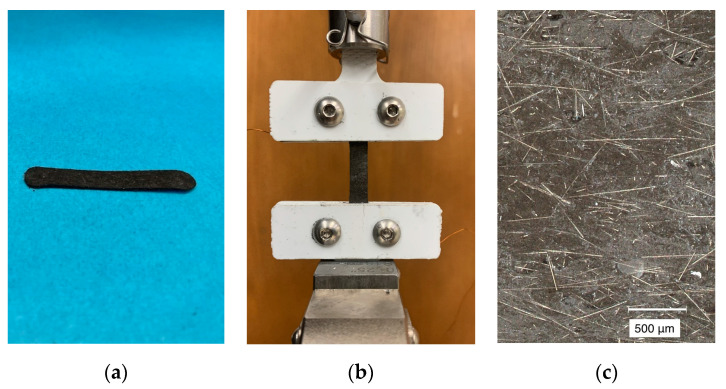
Depiction of (**a**) the high-deflection strain gauges; (**b**) the strain gauge during a tensile test; and (**c**) a microscopic image of the sensors under a small load. During tensile tests (**b**), the sensor was secured between two polypropylene grips. The grips contained a copper metal interior coating to facilitate measurement of the strain gauge’s electrical resistance at different loads.

In order to investigate how the strain-gauge resistance responded to different loads, a series of tensile tests were conducted using an Instron 3345 (see Figure 1). The Instron was used to apply precise loads to the sensors and provide measurements of the sensor force and elongation during the loads. Polypropylene grips were applied to the sensor at either end, restricting elongation to the inner portion of the strain gauges (with an initial length of 20 mm). All tensile tests were conducted at room temperature. A pretension of approximately 0.1 N was applied to the sensors prior to the start of each tensile test to avoid initial slack. The sensors’ electrical properties during these tests were also measured using a NIH 9215 data-acquisition device.

### 2.1. Model Development

Due to the viscoelastic nature of the strain gauges, the resistance is dependent on the load applied to the strain gauge and the time that elapses after the load has been applied, making the electrical behavior highly nonlinear. A series of tensile tests were therefore conducted to understand and interpret the responses of the sensors under different loading conditions and the time following the loading transition.

#### 2.1.1. Experimentation

The first tests investigated the mechanical strength of the sensors, specifically to determine the (potentially rate-dependent) linear region of the stress–strain diagram. Guedes found that above certain stress levels, a viscoelastic material’s response became highly intricate and difficult to model [7]. Our studies confirmed that beyond the linear region of the nano-composite strain gauges, the electrical output also becomes increasingly complex. The strain gauges were pulled at a constant rate until failure at three strain rates, −0.05 mm/s, 0.5 mm/s, and 5.0 mm/s, which encompassed the expected rates of human skin stretch during motion. The data from these tests were used to generate stress–strain diagrams. The (rate-dependent) elastic modulus of the sensors was estimated as the average derivative of stress with respect to strain within the first 10% strain. The linear region of the stress–strain diagram was calculated on the same basis as estimating 0.2%-yield strain in a typical material [5]. Subsequent analysis of the sensors’ electrical properties targeted strains within or near the linear region of the stress–strain diagram.

To observe the sensors’ responses at different elongations, we programed the Instron 3345 to apply incremental displacements of 0.5 mm (i.e., 2.5% strain increments). After each incremental strain, the sensors were held steady for a period of five seconds to allow initial recovery of stress and resistance. This process was repeated until the sensors reached an elongation of 15% strain, after which the sensors were incrementally relaxed in reverse order, at the same rate, back to their original length. This process was repeated for ten cycles. To account for the rate-dependency of the viscoelastic materials [16], the experiment was repeated at multiple strain rates as before: 0.05 mm/s, 0.5 mm/s, and 5.0 mm/s. Each test was conducted with three strain gauges at each strain rate.

#### 2.1.2. Quasi-Static Model

The resistance response of the sensors during deformation involved an initial spike, or overshoot, in resistance relating to the initial change in strain [16], followed by an exponential decay towards a steady-state asymptote. Hence, the strategy for relating the strain imposed on the gauge with the resultant sensor resistance was as follows:

1. Determine a functional relationship between sensor strain and sensor resistance that approximates the steady-state behavior, based upon tabulating and interpolating experimental data.
2. Incorporate the short-term resistance peaks (“spikes”) and subsequent decay into the model based on observations of how strain magnitude and strain rate affect resistance spike magnitude and decay rate.

The quasi-static model captured only the effect of strain (under approximately static conditions) on a sensor’s resistance. Electrical data were collected from the tensile tests at each incremental strain after transient electrical behaviors had dissipated for five seconds. Spline fit curves were implemented to interpolate between the data points $\epsilon_{i}$ and $R_{i}$ and achieve a relationship between strain and resistance (see Equation (1)).

(1) $$R=f\left(\epsilon \right)=spline\left(\epsilon_{i},R_{i},\epsilon \right)$$

As stated previously, sensor resistances operate on the principles of percolation theory and quantum tunneling. This implies that the resistance is highly dependent on the distribution of nanoparticles on a microscopic level [1]. During the manufacturing process, the concentration of nanoparticles embedded in the silicone matrices is approximately uniform. However, even slight variation in the distribution of nanoparticles can have significant effects [2]. The static-strain model f(ϵ) is therefore specific to each sensor. However, the general trends for all sensors are likely to be similar.

Gauge factor (or sensitivity factor) is defined as the relative change in resistance with respect to strain (see Equation (2)) [1]. High gauge factors provide higher strain resolution. The gauge factor was calculated by taking the derivative of the static model (Equation (1)) with respect to strain, dividing it by the sensor resistance at its unstretched length, and dividing the product by strain (see Equation (2)).

(2) $$GF=\frac{\left(\Delta R/R_{0}\right)}{\epsilon }=\frac{f^{\prime }\left(\epsilon \right)/f\left(0\right))}{\epsilon }$$

Previous studies have found that rubbers “exhibit an appreciable change in their mechanical properties resulting from the first extension” [17] due to the Mullins effect. Initial sensor tests indicate that the strain gauges’ electrical properties during preliminary strains also significantly differ from the electrical properties during later strains. Therefore, during cyclic stresses and relaxations, the first stress-and-relaxation cycle(s) served to precondition the strain gauge to pass any initial viscoelastic phenomena that caused inconsistent electrical behavior. In order to determine how many preconditioning cycles were necessary to achieve consistent electrical behavior, the steady-strain model (Equation (1)) was derived using data from the first, second, third, and fourth cyclic strain cycles. These models ($\hat{f}\left(\epsilon \right)$) were used to predict the steady-state strain–resistance relationship for the remainder of the ten stress–relaxation cycles. The mean average error (MAE) of the predictions was used to evaluate model performances and determine how many stress–relaxation cycles were necessary for preconditioning.

#### 2.1.3. Dynamic Model

The dynamic-strain model accounts for the transient spikes that were ignored in the steady-state strain model. Previous studies attributed these spikes to viscoelastic stress relaxation [16]. Every time the sensor is pulled or relaxed to a new strain, the internal sensor stresses cause a “change [in] the distance between several [conductive] nanomaterials” [1], resulting in a resistance spike. According to this explanation, the sensor’s electrical behavior under dynamic conditions is heavily influenced by the sensor’s viscoelastic mechanical properties.

**Figure 2:**
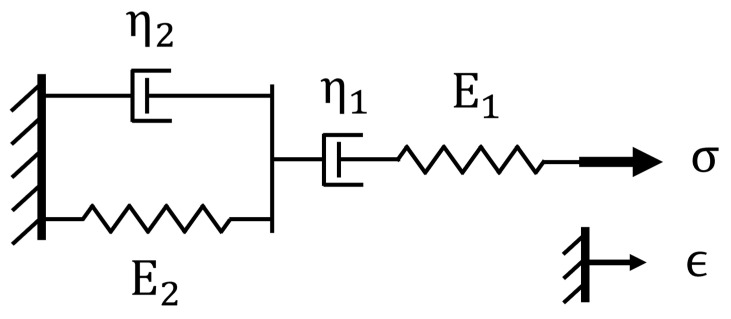
Burger’s rheological model of viscoelastic materials. The spring and damper in parallel ($E_{2}$ and $\eta_{2}$) capture the senor’s viscoelastic behavior, the spring in series ($E_{1}$) models the elastic behavior, and the damper in series ($\eta_{1}$) accounts for the viscoplastic behavior.

For a given applied macroscopic strain, the time-dependent distance between filler particles relates strongly to the stress localization in the soft polymer between the “hard” particles and the subsequent stress relaxation over time. Hence, the magnitude of the resistance spike is presumably inversely related to the increase in internal sensor stress. Time dependence of stress and strain in a typical polymer is commonly modeled via Burger’s rheological model (see Figure 2). This model predicts a rate-dependent increase in stress during application of the macro strain, followed by an approximately exponential decay during the stress relaxation phase. This causes the superposition of rate-dependent spikes and subsequent exponential decay in resistance on top of the quasi-static-resistance model. The data collected from the incremental strain tests were used to estimate the relative increase in resistance (S) following an incremental strain step under different strains and strain-rate conditions (e.g., if the sensor’s resistance under static conditions is 100 Ω and the sensor resistance, including the spike, is 200 Ω, the relative resistance spike magnitude, S, is equal to one).

During the application of a series of stepwise strain changes to the sensor, once the sensor reaches its new macroscopic strain, the internal stresses relax, causing the resistance spike to dissipate. Weibull’s equation describes viscoelastic strain recovery as an exponential decay transition [5]. Preliminary experimental data indicated that the transient mechanical and electrical behavior followed a similar dissipation rate. The time constant (λ) of this exponential decay was estimated from experimental data using MATLAB’s gradient-based optimization algorithm, *fminunc.* The criterion for the optimal λ was the predicted stress-relaxation response that minimized the sum of squared differences between the predicted stresses and the observed stresses. The λ constants were tabulated by strain rate and strain magnitude and were used to predict the rate of resistance-spike dissipation.

#### 2.1.4. Combined Model

Using the steady-state strain–resistance model (Equation (1)) and the viscoelastic-dependent parameters (S and λ), an equation was developed that predicts the sensor response during transitions between strain levels (see Equation (3)). The model is a function of the quasi-static-resistance–strain relationship (f(ϵ)), the resistance-spike magnitude (S), the stress–relaxation coefficient (λ), and the time since the previous resistance-spike occurrence ($t-t_{s}$).

(3) $$R=f\left(\epsilon \right)*\left[1+S\;e^{-\lambda \left(t-t_{s}\right)}\right]$$

### 2.2. Model Validation

The model was evaluated with a series of tensile tests, during which different strains and strain rates were applied to the sensors. The model’s performance was evaluated by calculating the r-squared correlation between the observed and predicted response, as well as the mean absolute error (MAE) of the model predictions. Three validation tests were conducted to assess the model accuracy.

- Quasi-static interpretation: The static-strain resistance model (Equation (1)) was used to interpret the sensor resistance under static conditions to predict the sensor strain.
- Sensor output predictions: The dynamic-strain resistance model (Equation (3)) was used to predict sensor resistance as a function of a known strain and strain rate.
- Biomechanical application: The dynamic-strain resistance model (Equation (3)) was used to interpret sensor strain and strain rate during a tensile test that imitates cyclical biomechanical function.

**Figure 3:**
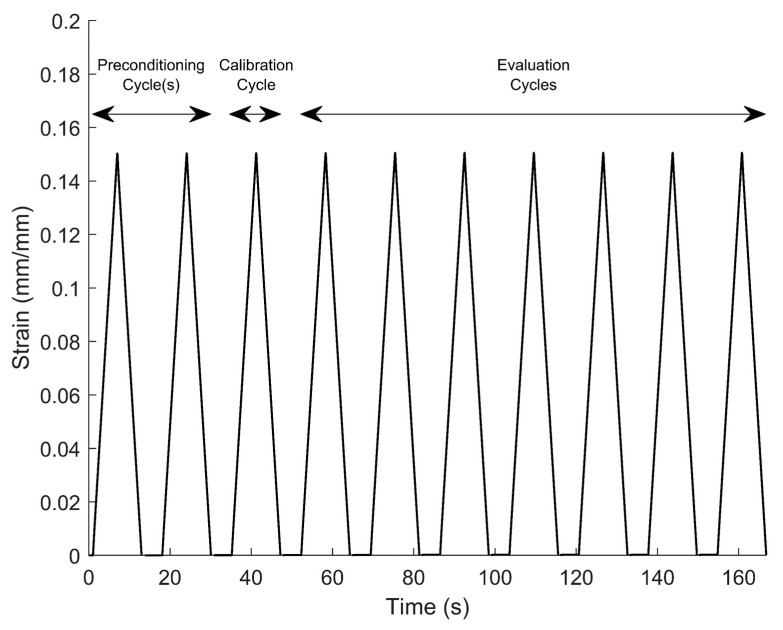
Depiction of the strain profile that the tensile tester applied to a sensor, and which cycles constituted preconditioning, calibration, and evaluation of stress relaxation.

As stated previously, the static-strain model (Equation (1)) for each sensor is unique due to the variation in the microscopic distribution of nanoparticles in the silicone matrix, and the resistance response during the first stress–relaxation iteration(s) is significantly different than during subsequent repetitions. The first cycle(s) therefore served to precondition the sensors. After preconditioning was complete, the steady-state strain model (Equation (1)) was calibrated by measuring the sensor resistance at 0% strain and 15% strain. The data point taken at 0% strain was collected after the transient spike had dissipated. The resistance spike at 15% strain had not yet dissipated when the resistance was measured. Instead, the steady-state strain resistance was estimated by dividing the sensor reading by (1+S). A template of the static-strain resistance was obtained from the incremental strain tensile tests, and it was scaled and shifted using the two data points to represent the static-strain response of the sensor of interest. The dynamic-model parameters (spike magnitude coefficients S and spike-decay coefficients λ) were obtained from the incremental strain experiments described above. The model was then used to analyze and interpret the output from the stress–relaxation cycles following the calibration cycle (see Figure 3).

#### 2.2.1. Quasi-Static Interpretation

The first validation test evaluated the model’s ability to interpret strains under approximately quasi-static conditions. After the sensors were preconditioned, and the static-strain model was developed as described above, the sensors were pulled to a series of random strains between 5 and 15% strain (strains were selected using a uniformly distributed random number generator). The sensors were held at each randomized strain for five seconds, and then relaxed back to the original length. The minimal resistance during the static period was extracted from each repetition and compared with the static-strain model (Equation (1)). This reading was used to interpret sensor strain. In total, ten stress–relaxation cycles were conducted (including preconditioning cycles). These tests were conducted at a strain rate of 0.5 mm/s and repeated with 10 strain gauges.

#### 2.2.2. Sensor-Output Predictions

The second validation test analyzed the model’s ability to predict strain-gauge resistance during a known strain and strain rate. Following preconditioning and calibration cycles, the sensors were pulled to 15% strain, immediately relaxed to initial length, and held for five seconds. This process was repeated until 10 stress–relaxation cycles were completed. Using the dynamic-strain model (Equation (3)) and the known strain and strain rates, the sensor resistance for the remaining stress–relaxation cycles was predicted. These tests were conducted at rates of 0.05 mm/s, 0.5 mm/s, and 5.0 mm/s. Three sensors were tested at each strain rate.

#### 2.2.3. Model Evaluation during Simulated Biomechanical Application

The intended purpose of high-deflection strain gauges is to monitor biomechanical motion. The final evaluation test analyzed the model’s ability to interpret elongations that would be typical during repetitive biomechanical activities. Random strain rates between 0.05 and 5.0 mm/s were selected for this evaluation (using a logarithmically distributed random number generator). Strain magnitudes for the analysis were selected between 5 and 15% strain (using a uniformly distributed number generator). The sensors were preconditioned (at a strain rate that was randomly selected) and calibrated using the methods described above to obtain the dynamic-strain model (Equation (3)). Subsequently, the sensors were repeatedly pulled to the randomly selected strain at the randomly selected strain rate. The dynamic-strain model was used to estimate the sensors’ strain magnitude by tracing the resistance values to the nearest strain. The strain rate was estimated by dividing the estimated strain magnitude by the time required to reach the maximum strain.

## 3. Results

### 3.1. Model

As shown in the strain-to-failure diagrams, it was found that the linear portion of the stress-strain curve did not significantly vary between strain rates (see Table 1).

These results provided us with an initial indication of the linear range of the stress-strain diagram. Subsequent characterization tests of the sensor’s electrical and mechanical properties were limited to strains near the linear stress–strain region.

#### 3.1.1. Quasi-Static Model

**Figure 4:**
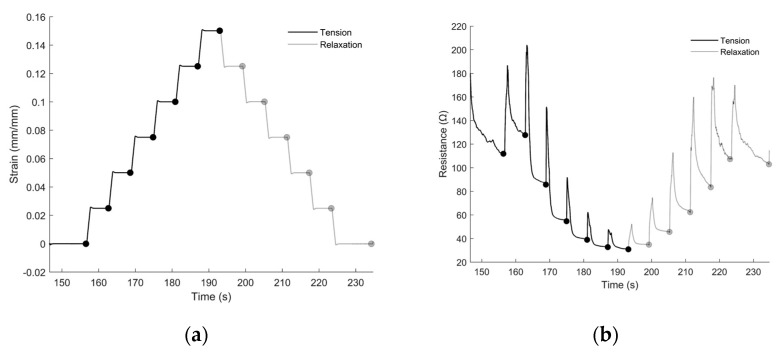

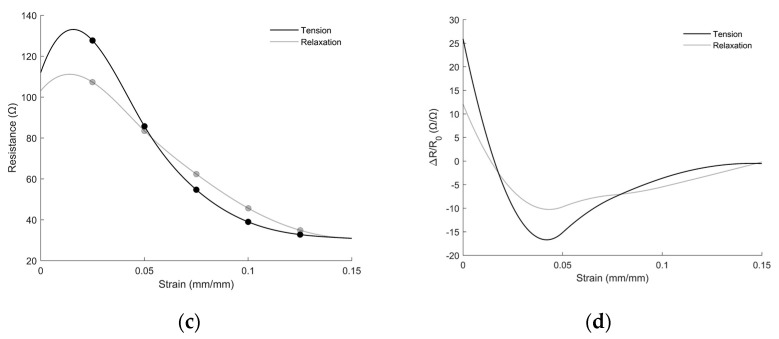
(**a**) The strain profile of an incremental strain test (black line indicates a sensor’s performances as it is pulled in tension, and the gray line indicates the sensor’s performance as it is relaxed; dots represent the points at which empirical data were collected from the resistance test after the transient spike had dissipated for all sub-figures). (**b**) The electrical response from the sensors over time as the strain gauges were pulled to different incremental strains. (**c**) The strain–resistance relationship interpolated from the tensile tests. (**d**) The gauge factor (relative change in resistance with respect to strain). Spline—fit curves were used to interpolate the resistance of the sensor under static conditions between the empirical points. The derivative of the spline-fit curves over the spline fits depicts the relative sensitivity of the sensor at different strains.

During the incremental strain tests, both the transient and the ‘steady-state’ (i.e., the post-spike) response of the sensors were observed. For the preliminary sensor model, we desired to capture only the effect of steady-state strains on the sensor’s electrical resistance. This was accomplished by collecting data points at each incremental strain level (Figure 4a) after the transient spike had dissipated (Figure 4b). The resulting stress–strain relationship and sensitivity factor are depicted in Figure 4c,d.

Using the resistance data points collected during static strains after the resistance spike had dissipated, we determined the strain–resistance relationship by interpolating between data points with polynomial fit curves (see Equation (4), Table 2).

(4) $$R=f\left(\epsilon \right)=c_{0}+c_{1}{\left(\epsilon -\epsilon_{0}\right)}^{1}+c_{2}{\left(\epsilon -\epsilon_{0}\right)}^{2}+c_{3}{\left(\epsilon -\epsilon_{0}\right)}^{3},\;\epsilon_{0}<\epsilon$$

The critical strain is defined as the point after which the sensor response becomes monotonic (the strain after which the resistance will only increase/decrease with additional strain) [4]. The critical strain for these sensors is approximately 1.5% strain.

The gauge factor is highly nonlinear. The optimal performance of the sensor occurs at approximately 7–8% strain, after which the magnitude of the gauge factor decreases.

**Figure 5:**
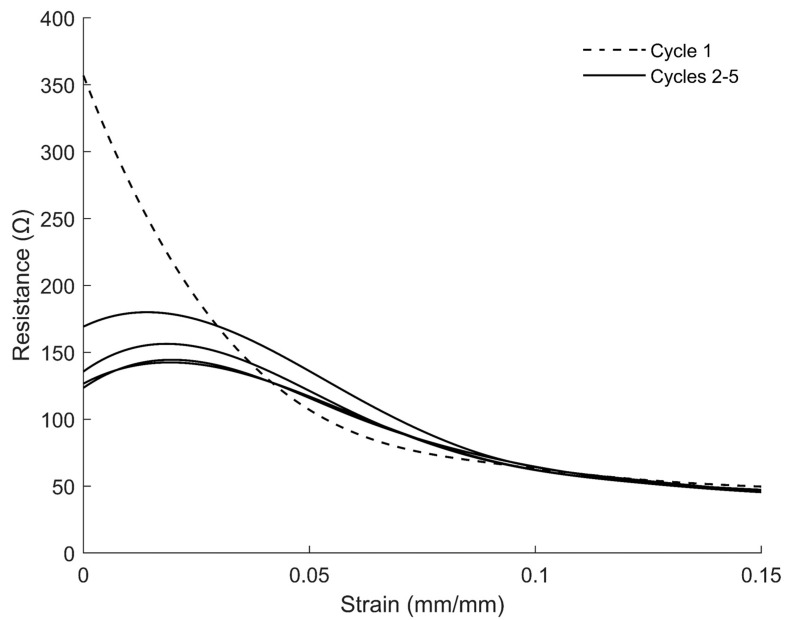
Depiction of the strain–resistance relationship during the first five stress–relaxation cycles.

We observed that the resistance of the sensors during the first cycles exhibited significantly different behavior compared with during the subsequent cycles due to primary creep (see Figure 5). The model is not intended to capture this highly transient behavior; hence, it is calibrated against data from a subsequent cycle. As stated previously, steady-state strain–resistance models $\hat{f}\left(\epsilon \right)$ (Equation (1)) were generated using data collected during the first, second, third, and fourth stress–relaxation cycles to predict the static-strain response of the sensor for the remainder of the ten stress–relaxations cycles. The resulting mean absolute errors (MAEs) are shown in Table 3.

It was observed that the MAE generally decreased with each additional preconditioning cycle. However, it was also desirable to develop the static-strain model with as few cycles as possible to maximize the predictive power of the model (e.g., if three preconditioning cycles were necessary, the model would be calibrated during the fourth stress–relaxation cycle and would only be capable of predicting the resistance of the sensor during the fifth stress–relaxation cycle and beyond). The largest reduction in error occurred between $\hat{f}{\left(\epsilon \right)}_{2}$ and $\hat{f}{\left(\epsilon \right)}_{3}$. The first two stress–relaxation cycles were therefore utilized for preconditioning the sensors, and the third cycle was used for calibrating the steady-strain resistance model described in Equation (1).

#### 3.1.2. Dynamic Model

**Figure 6:**
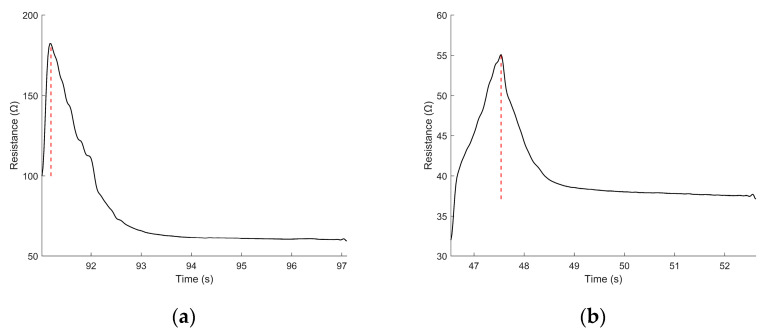
The sensor electrical response following (**a**) an incremental strain step of 0.5 mm and (**b**) following an incremental relaxation step of 0.5 mm. The solid black lines indicate the sensor resistance. The dotted red lines indicate the peak of the resistance spike during the transition between strains.

During each transition from one strain level to the next, the sensor exhibited a transient resistance spike. The spike magnitude was defined as the increase of resistance from its static-strain resistance (see Figure 6). From the incremental strain tests, the increase in resistance under each strain and strain-rate condition were obtained. The average S parameters are depicted in Table 4.

**Figure 7:**
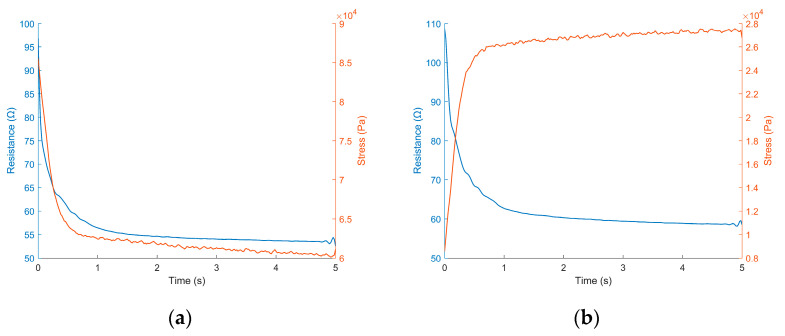
Stress–relaxation (orange) and transient resistance response (blue) during stress relaxation following an incremental step in tension (**a**) and following an incremental step in relaxation (**b**).

As expected, the strain changes at higher strain rates resulted in higher resistance spikes. As discussed earlier, if the resistance spike relates to localized and short-lived stress levels between conductive particles, these observations correlate with Burger’s rheological model relating stress to strain and strain rate in viscoelastic materials. Similarly, the stress–relaxation behavior of the sensors demonstrated a strong correlation with the resistance-spike dissipation (see Figure 7). This observation validated our hypothesis that both can be approximated as an exponential decay with time constant, λ, as in Weibull’s model, which captures the strain recovery of viscoelastic materials. The time constant, λ, was found to be both rate- and strain-dependent. The average λ values observed in the sensors under a variety of strains and strain rates are depicted in Table 5 (one outlier was discarded from the data).

#### 3.1.3. Combined Model

The aim of this study is to provide a model that relates measured resistance to skin strain during a set of cyclical exercises performed by a human subject. The quasi-static and dynamic components of the model described in the previous sections were combined into the final model through the following steps. For a given sensor, two preconditioning stress and relaxation cycles from 0 to 15% strain (at any strain rate) were applied to the sensors to mitigate Mullins effects, and a third stress-and-relaxation cycle was used to calibrate the sensor-specific model. The quasi-static model, Equation (1), was fitted to these data points to depict the relationship between static strains and resistance. For the initial sensor used in this study, the parameters of this model are given in Table 2; for a different sensor, the curve given by Equation (4) and Table 2 is scaled and shifted based upon two data points taken at strains of 0 and 0.15.

The parameters for the dynamic component of the model, Equation (3), were then obtained by incorporating the resistance-spike and exponential-decay-rate parameters (S and λ) from Table 4 and Table 5 (which remained constant for all the sensors made using the same method). Consequently, this model can be used to predict and interpret sensor output during any sequence of static and dynamic loading conditions.

### 3.2. Model Validation

#### 3.2.1. Quasi-Static Model Performance

**Figure 8:**
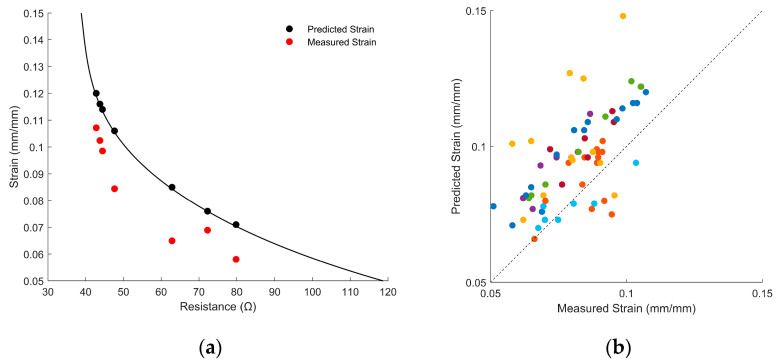
(**a**) The quasi-static strain model (black line), the model prediction at the randomly selected strain points (black dots), and the actual strains (red dots) for a single sensor. Vertical distances between black and red line represent the model error. (**b**) Scatterplot of the randomized strains (x-axis) compared to the model prediction (y-axis) for all 10 sensors (each strain gauge is indicated by a different color). Predictions that fall on the dotted black line are exactly accurate.

The model’s ability to interpret strains under static conditions was evaluated by preconditioning and calibrating 10 sensors. The strain gauges were pulled to a series of randomly selected sequenced strain changes, and the strain was estimated using the quasi-static model described above (Equation (1)); note that the original sensor used to calibrate the model was not included in the 10 test sensors. The quasi-static sensor model for a single sensor is depicted, along with its strain predictions and measured strains, in Figure 8a. The results from all ten sensors, comparing the randomly selected strain to the model’s estimation, are depicted in Figure 8b. The static-strain model evaluated achieved a MAE of 1.6% strain for all the sensors tested and an r-squared score of 0.64 (see Figure 8). It was observed, however, that the prediction error varied for each sensor—the MAE for each individual sensor ranged between 0.5 and 4.4% strain. The resistance measurements outside the model range were neglected. As can be seen in Figure 8a,b, the model tended to overestimate the magnitude of the quasi-static strains. It was observed that the quasi-static strain–resistance relationship generally drifted downward (i.e., the sensor would exhibit a lower resistance for a given strain than it had during prior strains at the same magnitude). This is consistent with observations in other investigations of viscoelastic sensors [18], and it was likely due to viscoplastic deformation, as described in Burger’s model.

#### 3.2.2. Dynamic Model’s Performance

**Figure 9:**
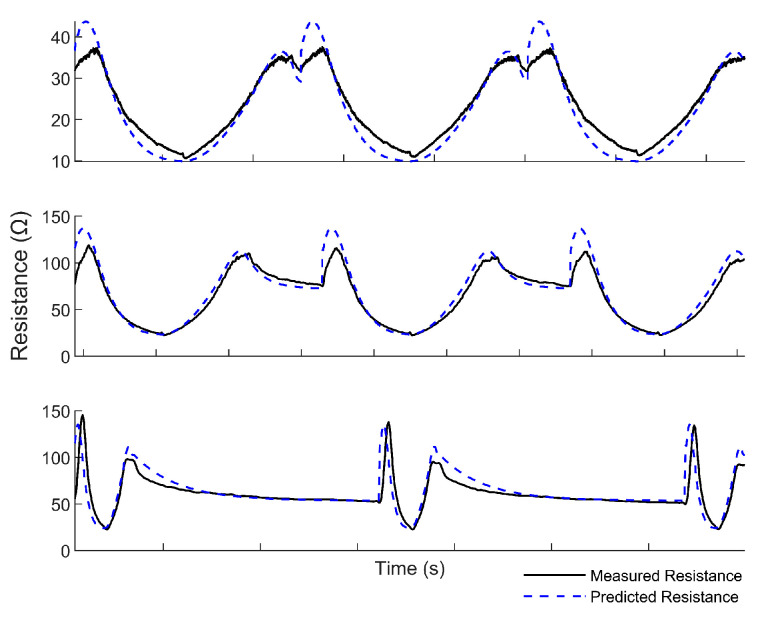
The predicted resistance value (dotted blue line) compared to the true sensor resistance (black line) during the validation test. The top plot is for validation conducted at a rate of 0.05 mm/s, the middle plot is the validation test conducted at a rate of 0.50 mm/s, and the bottom plot is the validation test conducted at a rate of 5.0 mm/s.

The full model was then used to predict the time-dependent strain from the resistance measured during the random sequence of strain changes mentioned in the previous section. When predicting the resistance values of sensors pulled from 0 to 15% strain at low-to-intermediate strain rates (0.5 mm/s or slower), the model achieved r-squared values greater than 0.9 (see Table 6). For all strain rates, the model captured the general trends in the resistance data exhibited by the viscoelastic sensors, but the MAE increased slightly for faster rates (Figure 9).

#### 3.2.3. Model Evaluation during Simulated Biomechanical Application

**Figure 10:**
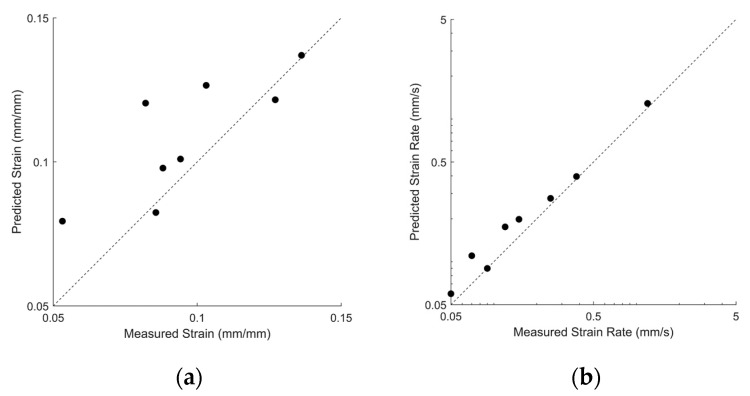
(**a**) Scatterplot of the true strain compared to the predicted model strain for different sensors, each pulled to a random strain (between 5 and 15% strain). (**b**) Scatterplot of the true strain rate of each sensor compared to the predicted strain rate, pulled at a random strain rate between 0.05 and 5.0 mm/s. Each sensor is represented by a single scatter point.

As stated previously, one of the most important potential applications of strain gauges is to provide estimations of biomechanics (e.g., range of motion, velocity, etc.). The sensors were therefore tested in a broad range of strains and strain rates to evaluate how the model interprets strain and strain rates during biomechanical activities. The strain gauges were preconditioned, calibrated, and then pulled to randomly selected strains (between 5 and 15%) at randomly selected strain rates (between 0.05 mm/s and 5.0 mm/s) on the Instron machine to imitate the range of expected strains and strain rates that a sensor would undergo during repetitious biomechanical applications. Ten sensors were evaluated during this application. The dynamic-strain model achieved a MAE of 1.4% strain when used to predict strain and a MAE of 0.036 mm/s when used to predict strain rate; the corresponding r-squared values for predicted strain magnitude and strain rate were 0.80 and 0.99, respectively (see Figure 10). Two sensors that exhibited resistance readings outside the range of the model were ignored.

## 4. Discussion

The present work investigates the output of high-deflection, resistance-based strain gauges. The findings from this research support those of previous works, which found that viscoelastic sensors exhibit highly nonlinear outputs [1,16,18]. However, this study also builds on these findings by quantifying the relationship between strain and resistance under quasi-static and dynamic conditions during cyclical strains. The quasi-static relationship between the resistance and strain of high-elongation viscoelastic sensors (Equation (1)) was determined by fitting an equation to the observed data of a single sensor. The model was subsequently calibrated for other sensors by taking two resistance data points at known strains and shifting the fitted curve to the new points. The dynamic relationship between the strain and the resistance, which was heavily influenced by the sensors’ viscoelastic properties, was then superimposed onto the quasi-static model to account for the resistance spikes and their subsequent dissipation (Equation (3)).

The resulting model was validated in a variety of tests to determine its ability to correctly interpret both the quasi-static and the dynamic strains of sensors from resistance measurements. When used to interpret static strains, the model achieved a MAE of 1.6% strain. The model prediction of the sensor output of dynamic-strain applications captured the general trends of the viscoelastic resistance behavior and achieved high accuracy at low-to-intermediate strain rates (MAE values of 5.00 Ω, 4.03 Ω, and 8.59 Ω at elongation rates of 0.05 mm/s, 0.50 mm/s, and 5.0 mm/s, respectively). Through the model’s interpretation of dynamic strains, it was possible to estimate strain magnitude with an MAE of 1.4% strain and strain rate with an MAE of 0.036 mm/s. These validation tests provide an estimate of the model’s ability to predict and interpret a wide range of strains and strain rates that encompass the expected sensor loads’ potential applications.

The intended purpose of the model is to interpret spinal biomechanics from an array of sensors adhered to a patient’s lower back. The findings from this endeavor will be valuable in the analysis of the strain-gauge outputs to extract biomechanical features from subjects’ spinal motions (e.g., range of motion, velocity, etc.). This innovative motion-monitoring technique may be used by clinicians to objectively assess patients’ spinal movement patterns. This, in turn, could be used to monitor movement changes and progress and to help identify the optimal treatment paradigms for millions of chronic low-back-pain patients worldwide. Additionally, these findings provide a foundation for interpreting all viscoelastic, resistance-based sensors, which can be used in other applications. 

Because this model was restricted to the linear region of the stress–strain diagram, where the strain gauges exhibited a predominately elastic and viscoelastic response, the effects of viscoplasticity on the electrical output were neglected. However, this model does account for nonlinear response with respect to load and time. Future iterations of the model could improve its performance by further quantifying the relationship between viscoplasticity and sensor resistance, which would become increasingly relevant to interpreting sensor outputs with additional stress-and-relaxation cycles (i.e., with additional stress-and-relaxation cycles, the effects of viscoplastic creep become more pronounced in the sensor output).

Future research can also be conducted to fine-tune the sensor recipe to achieve desired material properties and model parameters (e.g., reducing the nanoparticles to expedite resistance-spike dissipation). Additional investigations into preconditioning methods may lead to more effective methods and improved model performance.

There were slight instrumentation errors during the tensile tests at high strain rates (the incremental strain tests pulled the sensors by increments of 0.6 mm rather than 0.5 mm). This may have affected the specific parameters used for estimating the sensor resistance tested at a strain rate of 5.0 mm/s. However, the general trends observed, the model’s performance, and the conclusions regarding the effect of viscoelasticity on the strain gauge’s electrical output are still valid for the resistance-based sensors.

## Figures and Tables

**Table 1:** The average yield strain from sensors pulled at 5.0 mm/s, 0.5 mm/s, and 0.05 mm/s. Note that the linear strain range of the stress–strain diagram was not significantly affected by strain rate.

Strain Rate (mm/s)	Linear Strain (Percent)
5.00	11.26
0.50	12.35
0.05	12.41

**Table 2:** Coefficients for Equation (4) to describe the strain–resistance relationship during tension.

$\epsilon_{0}$	$c_{0}$	$c_{1}$	$c_{2}$	$c_{3}$
0	1.468 × 10^6^	−1.793 × 10^5^	4.520 × 10^3^	1.383 × 10^2^
0.025	1.468 × 10^6^	−6.906 × 10^4^	−1.697 × 10^3^	1.622 × 10^2^
0.05	3.629 × 10^5^	4.120 × 10^4^	−2.394 × 10^3^	9.940 × 10^1^
0.075	−6.710 × 10^4^	1.399 × 10^4^	−1.014 × 10^3^	5.963 × 10^1^
0.10	−8.356 × 10^4^	8.941 × 10^3^	−4.400 × 10^2^	4.194 × 10^1^
0.125	−8.356 × 10^4^	2.674 × 10^3^	−1.496 × 10^2^	3.522 × 10^1^

**Table 3:** The steady-state strain–resistance model, Equation (1), was estimated using the first, second, third, and fourth cycles. The resulting mean-squared error of this model (in Ohms) was used to determine how many cycles were needed for preconditioning purposes.

**Strain Rate (mm/s)**	$\hat{f}{\left(\epsilon \right)}_{1}\;\mathrm{MAE}\;(\Omega )$	$\hat{f}{\left(\epsilon \right)}_{2}\;\mathrm{MAE}\;(\Omega )$	$\hat{f}{\left(\epsilon \right)}_{3}\;\mathrm{MAE}\;(\Omega )$	$\hat{f}{\left(\epsilon \right)}_{4}\;\mathrm{MAE}\;(\Omega )$
0.05	8.51	8.50	5.69	4.64
0.5	17.92	15.18	7.38	5.05
5.0	16.79	11.16	6.40	6.51

**Table 4:** The spike magnitude, S (in units of Ω/Ω), was found to be dependent on strain rate. At higher strain rates, the magnitude of the resistance spikes increased. Note that at low strains, the spike magnitudes are significantly smaller, most likely due to sensor buckling.

Rate (mm/s)	Strain (Percent)						
	0	2.5	5	7.5	10	12.5	15
0.05	0.182	0.187	0.263	0.319	0.230	0.264	0.214
0.5	0.429	0.534	0.673	0.785	0.700	0.566	0.449
5.0	0.915	0.981	1.192	1.358	1.173	0.899	0.751

**Table 5:** The time constant, λ (in units of s^−1^), was found to be rate- and strain-dependent. At higher strains and strain rates, the sensors exhibited faster transient responses. Note that at low strains, the stress–relaxation constants are significantly lower, possibly due to strain-gauge buckling.

Rate (mm/s)	Strain (Percent)						
	0	2.5	5	7.5	10	12.5	15
0.05	0.433	0.718	0.778	0.804	0.818	0.815	0.736
0.5	0.875	1.914	1.979	2.011	2.089	2.220	1.641
5.0	1.121	4.390	3.178	4.051	4.176	4.231	3.391

**Table 6:** Model application test results (r-squared and mean-averaged error) conducted at different strain rates.

Strain Rate (mm/s)	R^2^	MAE (Ω)
0.05	0.96	5.00
0.50	0.96	4.03
5.00	0.80	8.59
